# Draft Genome Sequence of *Anaeromyxobacter* sp. Strain PSR-1, an Arsenate-Respiring Bacterium Isolated from Arsenic-Contaminated Soil

**DOI:** 10.1128/genomeA.00472-15

**Published:** 2015-05-14

**Authors:** Mimori Tonomura, Ayaka Ehara, Haruo Suzuki, Seigo Amachi

**Affiliations:** a Graduate School of Horticulture, Chiba University, Matsudo City, Chiba, Japan; b Graduate School of Science and Engineering, Yamaguchi University, Yamaguchi, Japan

## Abstract

Here, we report a draft genome sequence of *Anaeromyxobacter* sp. strain PSR-1, an arsenate-respiring bacterium isolated from arsenic-contaminated soil. It contained three distinct arsenic resistance gene clusters (*ars* operons), while no respiratory arsenate reductase gene (*arr*) was identified.

## GENOME ANNOUNCEMENT

*Anaeromyxobacter dehalogenans* is a facultative anaerobic myxobacterium within the *Deltaproteobacteria* and is able to utilize a wide variety of electron acceptors, including halogenated phenols, nitrate, nitrous oxide, fumarate, oxygen, Fe(III), U(VI), and Se(IV) (1–5). The metabolic versatility of *A. dehalogenans* makes it a promising candidate for bioremediation, such as the *in situ* biostimulation of U(VI) immobilization (6). Previously, we isolated a novel arsenate-respiring bacterium, designated strain PSR-1, from arsenic-contaminated soil (7). The strain was phylogenetically closely related to *A. dehalogenans* 2CP-1^T^ with 16S rRNA gene similarity of 99.7% and utilized not only arsenate but also nitrate, fumarate, oxygen, and Fe(III) as electron acceptors. It is widely accepted that arsenate-respiring bacteria play an important role in arsenic release from anoxic sediments in the form of arsenite (8). To the best of our knowledge, strain PSR-1 is the first *Anaeromyxobacter* species known to respire arsenate. Therefore, the draft genome sequence of strain PSR-1 may improve our understanding of this genus and its potential role in the biogeochemical cycling of arsenic in the environments.

Strain PSR-1 was grown anaerobically with fumarate as the electron acceptor and DNA was extracted using a DNeasy blood and tissue kit (Qiagen, Hilden, Germany). The draft genome sequence of PSR-1 was prepared using the Roche 454-FLX Titanium, and the sequencing reads were assembled using the Roche GS *de novo* Assembler (Newbler v. 2.8). After removal of sequences with low sequencing depth, the resulting assembly contains 344 contigs consisting of 4,863,754 bp, with a G+C content of 74.4%. Genome annotation was performed using Prokka 1.9 (9), yielding a total of 4,484 protein-coding genes. Putative gene functions were assigned using public databases, including COG (10), KEGG (11), and Pfam (12).

The genome contains three distinct arsenic resistance gene clusters (*ars* operons), each of which includes a detoxifying arsenate reductase gene (*arsC*). One consists of genes for a regulatory protein (*arsR*), an arsenite efflux pump (*arsB*), a pump-driving ATPase (*arsA*), and a chaperone of the pump (*arsD*) (*arsD*-*arsA*-*uspA*-*arsR*-*arsB*-*arsC*-*arsD*-ORFs-*arsA*). The second one consists of similar genes as well as an arsenite methyltransferase gene (*arsM*) (*arsD*-*arsC*-*uspA*-*arsB*-*arsR*-*arsM*). Respiratory arsenate reductase genes (*arrAB*) were not identified, despite a unique characteristic of this strain to respire arsenate. The draft genome contains at least 64 genes coding for *c*-type cytochromes, among which 54 are multiheme cytochromes. A wide variety of genes for type IV pilus assembly proteins (*pil* genes), flagellar production (*fli* and *flg* genes), adventurous motility (*agl* and *agm* genes), and chemotaxis proteins (*che* genes) are identified. Genes for antioxidant proteins, including superoxide dismutase, thiol peroxidase, alkyl hydroperoxide reductase, and rubrerythrin are found, but a catalase-peroxidase gene is absent. A reductive dehalogenase gene is absent. A nearly complete set of genes for the reductive acetyl-CoA pathway was found, except for the formate-tetrahydrofolate ligase gene, but there are no other autotrophic CO_2_ fixation pathway genes.

### Nucleotide sequence accession numbers. 

The PSR-1 whole-genome shotgun project has been deposited at DDBJ/EMBL/GenBank under the accession number BAZG00000000. The version described in this paper is the first version, BAZG01000000 (BAZG01000001 to BAZG01000344).
